# Association of low testosterone with changes in non-cardiovascular biomarkers in adult men

**DOI:** 10.1038/s41443-019-0112-4

**Published:** 2019-01-22

**Authors:** Michael Samoszuk, Abraham Morgentaler, Mark de Groot, Wouter van Solinge, Yu Li, Fiona Adair, Imo Hoefer, Saskia Haitjema

**Affiliations:** 1 1 Serna, Rancho Santa Margarita, CA 92688 USA; 2 Men’s Health Boston, Beth Israel Deaconess Medical Center, Harvard Medical School, 1200 Boylston St, Chestnut Hill, MA 02467 USA; 3 UMC Utrecht, Laboratory of Clinical Chemistry and Hematology, G03.550, Heidelberglaan 100, 3584CX Utrecht, Netherlands; 4 UMC Utrecht, Laboratory of Clinical Chemistry and Hematology, G03.550, Heidelberglaan 100, 3584CX Utrecht, Netherlands; 5 Beckman Coulter, Inc., 250S. Kraemer Blvd, Brea, CA 92821 USA; 6 Beckman Coulter, Inc., 250S. Kraemer Blvd, Brea, CA 92821 USA; 7 UMC Utrecht, Laboratory of Clinical Chemistry and Hematology, G03.550, Heidelberglaan 100, 3584CX Utrecht, Netherlands; 8 UMC Utrecht, Laboratory of Clinical Chemistry and Hematology, G03.550, Heidelberglaan 100, 3584CX Utrecht, Netherlands

**Keywords:** Diagnostic markers, Gonadal disorders

## Abstract

Testosterone has effects on many organs and systems. The purpose of this study was to test the hypothesis that low testosterone is associated with changes in various non-cardiovascular biomarkers in men older than 40 who were tested for possible hypogonadism. We extracted data from 9939 outpatient men who were over 40 years old (median age 56) and who also had concurrent laboratory measurements of total testosterone and one or more biomarkers of interest: estradiol, uric acid, prostate-specific antigen (PSA), sex-hormone binding globulin (SHBG), luteinizing hormone, creatinine, bone alkaline phosphatase (BAP), creatine kinase, hemoglobin A1c, and 25-hydroxy-vitamin D, and body mass index (BMI). In a smaller exploratory study of 19 otherwise healthy men presenting for evaluation of possible hypogonadism, pre-albumin (a.k.a.transthyretin, a marker of anabolism) and testosterone were measured. Men with lower levels of testosterone had significantly (*p* < 0.001) lower mean levels of PSA, SHBG, luteinizing hormone, and estradiol. Overall, men with low levels of testosterone also had significantly (*p* < 0.001) higher mean levels of LDH and BAP, but these associations varied between men who were younger or older than 56 years. There was a moderate but statistically significant positive correlation (*r* = 0.63, *p* < 0.05) between testosterone levels and pre-albumin. These results confirm our hypothesis that testosterone deficiency is associated with a broad range of systemic changes demonstrable in hormonal and non-hormonal serum assays in men over 40 years old being tested for possible hypogonadism.

## Introduction

A recent study has demonstrated that low plasma testosterone is associated with elevated cardiovascular disease biomarkers [1]. Testosterone is also known to have effects on non-cardiovascular organs and systems, such as prostate, bone marrow, bone turnover, muscle, and metabolism [2, 3]. However, there is little published information available regarding possible associations between low levels of testosterone and changes in non-cardiovascular biomarkers that measure the functions and health of the organs and systems that are influenced by testosterone or its metabolites [4, 5]. Such associations, if they could be demonstrated to exist, might provide important insights into the physiological changes that accompany low testosterone. They might also have practical value in diagnosing or evaluating men presenting with possible late onset hypogonadism. Therefore, the primary purpose of the retrospective portion of this study was to test the hypothesis that low testosterone is associated with changes in non-cardiovascular biomarkers that are sometimes measured as part of the routine health assessment of men over the age of 40 who are being tested for possible adult onset hypogonadism.

Transthyretin (also known as pre-albumin) is one of the major proteins in the blood. It is produced by the liver, and its major functions are to transport thyroxin and vitamin A [6]. Although pre-albumin is mostly used as a marker of malnutrition in hospitalized or post-surgical patients, there is evidence that lower levels of pre-albumin are associated with a catabolic state while higher levels are associated with anabolic conditions [7–10]. Because testosterone is anabolic, a secondary objective of this study was to test the hypothesis in a small exploratory sub-study that there is an association between levels of pre-albumin and testosterone in otherwise healthy men who are being evaluated for possible adult onset hypogonadism.

## Materials and methods

The Utrecht Patient Oriented Database (UPOD) contains all health care data and measurements from all inpatients and outpatients treated or evaluated at the University Medical Center Utrecht in the Netherlands, which was the site of the Institutional Review Board that approved this study [11] From UPOD, we extracted data from 9939 male outpatients over 40 years old who had a laboratory measurement of total testosterone levels as well as a measurement of one or more of the following biomarkers on the same day: free testosterone, uric acid, estradiol, prostate-specific antigen (PSA), sex-hormone binding globulin (SHBG), luteinizing hormone (LH), creatinine, bone alkaline phosphatase (BAP), creatine kinase, lactate dehydrogenase (LDH), hemoglobin A1c, 25-hydroxy-vitamin D, and body mass index. These markers were selected for inclusion in this study, because they relate to organs or functions impacted by testosterone and also because they were also the most likely tests to be routinely ordered as part of the general medical workup of adult men who are being tested for possible hypogonadism.

All tests were performed on AU5800/DxI^®^ analyzers (Beckman Coulter, Inc., Brea, CA USA). The 95% reference range for total testosterone measured on the DxI analyzer is 175–781 ng/dL and is substantially lower than the reference ranges established for use by other analyzers from other manufacturers or thresholds for diagnosing low testosterone [12]. The reasons for the variations in reference ranges for testosterone between various manufacturers and assays have been described [13, 14]. The Beckman Coulter reference range was initially established by measuring testosterone levels in American men between the ages of 18 and 66 years of age, not otherwise specified. It was then validated at Utrecht for use with their patient population. We therefore believe that the 95% reference range was appropriate for use in this study. Nevertheless, it is possible that using a higher threshold for distinguishing between low and “normal” (or even optimal) levels of testosterone might yield more significant associations between testosterone and non-cardiovascular biomarkers. Free testosterone was calculated using the Vermeulen equation.

Analyses of the biomarker levels were stratified based on serum testosterone levels classified into lowest (<130 ng/dL), low (130–202 ng/dL), and normal (>= 202 ng/dL). These categories were not chosen arbitrarily. The threshold of 202 ng/dL is the clinical lower threshold for hypotestosteronism that was established at University Medical Center Utrecht. Thereafter, the lower group was split on the median to yield the “lowest” and “low” categories. Differences between testosterone strata were then assessed with the Kruskal–Wallis test.

The pre-albumin exploratory sub-study was a single-center (Boston Men’s Health Clinic, Boston, MA), observational, prospective, cohort study that enrolled men between the ages of 40 and 75 who presented with signs or symptoms suggestive of low testosterone but with no evidence of any other serious illness. Inclusion criteria were as follows:

- Written informed consent obtained
- Completion of standard questionnaires regarding sexual health
- Male sex at birth ≥40 and ≤75 years of age
- Presentation to the clinic with symptoms suggestive of low testosterone, such as loss of libido, erectile dysfunction, cognitive or mood disturbances, etc.
- Able and willing to provide blood specimens and follow study schedule

Exclusion criteria were:

- Previous exposure to exogenous testosterone, DHEA, clomiphene citrate, or other Selective Estrogen Receptor Modulators, or over-the-counter medications or herbals (Treatment Naive) intended to elevate total testosterone
- Use of opioid medication within 3 months prior to enrollment
- Serious psychiatric disease or uncontrolled medical illness (including serious inflammatory diseases) as suspected from medical history or clinical examination
- Use of any sex hormones or steroidal anabolic drug supplements (OTC or prescribed) (Treatment Naive)
- Incapable of giving informed consent or complying with protocol or unwilling to comply with protocol requirements
- Diagnosis of prolactinoma

A single blood specimen was obtained from 19 eligible men before any treatment. After the samples were clotted for 2 hours, the serum was removed, split into fractions, and frozen at −70 °C. The frozen samples were shipped to ARUP Laboratories (Salt Lake City, UT) where pre-albumin and testosterone were measured on a Beckman Coulter UniCel^®^ DxC System. The correlation coefficient (*r*-value) between pre-albumin and testosterone was then calculated using the CORREL function in Microsoft Excel. A *P*-value for the Pearson R-score was then calculated using an online calculator available at Social Science Statistics web site (https://www.socscistatistics.com/pvalues/pearsondistribution.aspx).

The clinical trial was registered at clinicaltrials.gov as NCT03313635. The committee that approved this study was the Beth Israel Hospital Institutional Review Board. Informed consent was obtained from all subjects.

## Results

Table 1 summarizes the overall results of all available measurements in the UPOD study for hormonal and non-hormonal biomarkers in all of the men, separated into three groups with lowest, low, and normal (high) testosterone levels. We considered vitamin D to be a hormone because of its physiologic functions and chemical similarities to testosterone. Measurements of biomarkers were available in more than 100 men for all biomarkers except for myoglobin, CK, and free PSA.

**Table 1:** Median values, interquartile ranges (IQR), and [*n*] of available measurements of hormonal and non-hormonal biomarkers in all men >= 40 years of age, stratified by testosterone levels

Hormonal biomarkers	Lowest testosterone <4.5 nmol/L (<130 ng/dL) [*n* = 574]	Low testosterone 4.5–7 nmol/L (130–202 ng/dL) [*n* = 763]	Normal testosterone >= 7 nmol/L (>202 ng/dL) [*n* = 8602]	*P-*value*
25-hydroxy-vitamin D (nmol/L)	54 (31–63) [*n* = 52]	48 (39–68) [*n* = 31]	55 (40–75) [*n* = 540]	0.08 [*n* = 623]
Estradiol (pmol/L)	38 (29–50) [*n* = 24]	57 (45–75) [*n* = 45]	90 (67–110) [*n* = 864]	<0.001 [*n* = 933]
Free testosterone (pmol/L)	59 (21–88) [*n* = 227]	140 (120–170) [*n* = 379]	310 (240–400) [*n* = 4962]	<0.001 [*n* = 5568]
LH (IU/L)	2.0 (1.0–6.0) [*n* = 226]	3.2 (1.8–6.6) [*n* = 275]	3.6 (2.4–5.8) [*n* = 3278]	<0.001 [*n* = 3779]
SHBG (nmol/L)	30 (19–37) [*n* = 280]	24 (17–28) [*n* = 430]	35 (25–39) [*n* = 5561]	<0.001 [*n* = 6271]
*Non-hormonal biomarkers*				
Age (years)	59 (51–70) [*n* = 574]	58 (50–67) [*n* = 763]	56 (49–65) [*n* = 8602]	<0.001 [*n* = 9939]
BAP (U/L)	79 (68–96) [*n* = 159]	80 (66–101) [*n* = 193]	74 (62–92) [*n* = 2659]	<0.001 [*n* = 3011]
BMI (kg/m^2^)	27.6 (25.0–30.5) [*n* = 59]	27.8 (25.3–31) [*n* = 79]	26.3 (23.9–29.4) [*n* = 737]	0.004 [*n* = 875]
CK (U/L)	153 (100–183) [*n* = 3]	226 (170–302) [*n* = 6]	120 (83–167) [*n* = 86]	0.05 [*n* = 95]
Creatinine (µmol/L)	89 (75–103) [*n* = 359]	93 (82–106) [*n* = 444]	90 (80–101) [*n* = 5327]	0.002 [*n* = 6130]
Free PSA (µg/L)	0.99 (0.64–1.35) [*n* = 2]	1.55 (1.53–1.58) [*n* = 2]	0.31 (0.20–0.87) [*n* = 50]	0.16 [*n* = 54]
HBA1c (mmol/mol)	40 (37–48) [*n* = 126]	40 (37–49) [*n* = 169]	39 (36–43) [*n* = 1828]	<0.001 [*n* = 2123]
LDH (U/L)	216 (180–314) [*n* = 110]	213 (184–293) [*n* = 168]	198 (171–254) [*n* = 2345]	<0.001 [*n* = 2623]
Myoglobin (µg/L)	[*n* = 0]	[*n* = 0]	59 (50–75) [*n* = 3]	Not applicable
PSA (µg/L)	0.64 (0.19–1.85) [*n* = 100]	0.90 (0.50–1.60) [*n* = 175]	0.95 (0.55–1.90) [*n* = 2457]	<0.001 [*n* = 2732]
Uric acid (mmol/L)	0.39 (034–0.46) [*n* = 9]	0.38 (0.35–0.44) [*n* = 19]	0.34 (0.29–0.41) [*n* = 263]	0.02 [*n* = 291]

*Kruskall–Wallis test

Also presented in Table 1 are the median values and interquartile ranges of all of the biomarkers in all of the men in the study. Compared to men with normal levels of testosterone, men with lower and lowest levels had significantly lower free testosterone, PSA, SHBG, LH, and estradiol. Age, BMI, and levels of LDH, BAP, hemoglobin A1c and uric acid were significantly (*p* < 0.05) higher in men with lowest and lower levels of testosterone compared to men with normal levels; however, the magnitudes of the differences between the groups were relatively small for hemoglobin A1c and uric acid.

In men younger than 56 years of age (Table 2), there was no significant association between age or BMI or LDH and testosterone levels. There were, however, significant associations between lower testosterone and lower free testosterone, creatinine, estradiol, LH, PSA, and SHBG. In these younger men, there was also a significant association between lower testosterone and higher levels of hemoglobin A1c as well as BAP.

**Table 2:** Median values, (IQR), and [*n*] of available measurements of biomarkers in men <56 years of age separated into three groups with lowest, low, and normal testosterone

Hormonal biomarkers	Lowest testosterone <4.5 nmol/L (<130 ng/dL) [*n* = 247]	Low testosterone 4.5–7 nmol/L (130–202 ng/dL) [*n* = 338]	Normal testosterone >= 7 nmol/L (>202 ng/dL) [*n* = 4148]	*P-*value*
25-hydroxy-vitamin D (nmol/L)	59 (37–66) [*n* = 23]	45 (35–72) [*n* = 16]	55 (38–76) [*n* = 264]	0.70 [*n* = 303]
Estradiol (pmol/L)	36 (32–43) [*n* = 8]	60 (53–77) [*n* = 13]	90 (69–110) [*n* = 299]	<0.001 [*n* = 320]
Free testosterone (pmol/L)	62 (22–90) [*n* = 109]	151 (130–180) [*n* = 186]	330 (260–430) [*n* = 2592]	<0.001 [*n* = 2887]
LH (IU/L)	1.6 (0.8–4.2) [*n* = 105]	2.7(1.6–5.1) [*n* = 147]	3.4 (2.3–5.3) [*n* = 1932]	<0.001 [*n* = 2184]
SHBG (nmol/L)	25 (17–26) [*n* = 135]	20 (14–26) [*n* = 206]	31 (22–41) [*n* = 2851]	<0.001 [*n* = 3192]
*Non-hormonal biomarkers*				
Age (years)	49 (44–53) [*n* = 247]	49 (45–52) [*n* = 338]	48 (45–52) [*n* = 4148]	0.17 [*n* = 4733]
BAP (U/L)	85 (70–102) [*n* = 60]	78 (68–101) [*n* = 82]	74 (62–90) [*n* = 1248]	<0.001 [*n* = 1390]
BMI (kg/m^2^)	28 (25–30) [*n* = 28]	28 (25–31) [*n* = 25]	27 (24–29) [*n* = 363]	0.16 [*n* = 416]
CK (U/L)	47 (47–47) [*n* = 1]	277 (168–310) [*n* = 5]	124 (88–172) [*n* = 52]	0.04 [*n* = 58]
Creatinine (µmol/L)	80 (70–95) [*n* = 147]	86 (77–97) [*n* = 168]	88 (79–97) [*n* = 2457]	<0.001 [*n* = 2772]
Free PSA (µg/L)	0.28 (0.28–0.28) [*n* = 1]	—	0.22 (0.13–0.31) [*n* = 32]	0.87 [*n* = 33]
HBA1c (mmol/mol)	41 (67–49) [*n* = 52]	41 (37–47) [*n* = 69]	37 (34–42) [*n* = 690]	<0.001 [*n* = 811]
LDH (U/L)	214 (178–272) [*n* = 49]	218 (183–298) [*n* = 76]	198 (170–270) [*n* = 1186]	0.06 [*n* = 1311]
Myoglobin (µg/L)	—	—	50 (46–55) [*n* = 2]	Not applicable
PSA (µg/L)	0.41 (0.178–0.66) [*n* = 32]	0.62 (0.40–1.03) [*n* = 60]	0.70 (0.49–1.10) [*n* = 1119]	<0.001 [*n* = 1211]
Uric acid (mmol/L)	0.39 (0.39–0.48) [*n* = 5]	0.37 (0.35–0.38) [*n* = 7]	0.34 (0.28–0.39) [*n* = 101]	0.08 [*n* = 113]

*Kruskall–Wallis test

By contrast, in men who were >= 56 years of age (Table 3), there were significant associations between lower testosterone levels and higher age, BMI, LDH, or creatinine. Interestingly, in this age group men with normal testosterone also had significantly higher levels of vitamin D. The associations between lower testosterone and lower estradiol, free testosterone, LH, SHBG, and PSA were the same in both age groups. Unlike the younger age group, however, there was no significant association between testosterone levels and hemoglobin A1c or BAP in the older group of men.

**Table 3:** Median values, (IQR) and [*n*] of available measurements of biomarkers in men >= 56 years of age separated into three groups with lowest, low, and normal testosterone

Hormonal biomarkers	Lowest testosterone [*n* = 327]	Low testosterone [*n* = 425]	Normal testosterone [*n* = 4454]	*P-*value*
25-hydroxy-vitamin D (nmol/L)	41 (31–58) [*n* = 29]	50 (43–58) [*n* = 15]	55 (40–75) [*n* = 276]	0.03 [*n* = 320]
Estradiol (pmol/L)	40 (29–50) [*n* = 16]	56 (45–75) [*n* = 32]	90 (66–112) [*n* = 565]	<0.001 [*n* = 613]
Free testosterone (pmol/L)	59 (20–86) [*n* = 118]	130 (110–160) [*n* = 193]	290 (230–370) [*n* = 2370]	<0.001 [*n* = 2681]
LH (IU/L)	2.8 (1.2–9.4) [*n* = 121]	3.8 (2.3–8.5) [*n* = 128]	4.0 (2.6–6.5) [*n* = 1346]	0.04 [*n* = 1595]
SHBG (nmol/L)	33 (21–52) [*n* = 1]	29 (20–41) [*n* = 2]	39 (29–52) [*n* = 18]	<0.001 [*n* = 21]
*Non-hormonal biomarkers*				
Age (years)	68 (62–75) [*n* = 327]	66 (61–72) [*n* = 425]	65 (60–71) [*n* = 4454]	<0.001 [*n* = 5206]
BAP (U/L)	78 (67–94) [*n* = 99]	81 (65–103) [*n* = 111]	75 (62–93) [*n* = 1411]	0.11 [*n* = 1621]
BMI (kg/m^2^)	28 (25–31) [*n* = 31]	28 (25–31) [*n* = 55]	26 (24–29) [*n* = 374]	<0.001 [*n* = 460]
CK (U/L)	183 (168–197) [*n* = 2]	175 (175–175) [*n* = 1]	105 (83–155) [*n* = 34]	0.18 [*n* = 37]
Creatinine (µmol/L)	94 (82–113) [*n* = 212]	96 (86–112) [*n* = 276]	92 (81–107) [*n* = 2870]	0.001 [*n* = 3358]
Free PSA (µg/L)	1.7 (1.7–1.7) [*n* = 327]	1.6 (1.5–1.6) [*n* = 425]	1.3 (0.9–1.7) [*n* = 4454]	0.48 [*n* = 5206]
HBA1c (mmol/mol)	40 (37–47) [*n* = 74]	40 (37–49) [*n* = 100]	40 (37–44) [*n* = 1138]	0.27 [*n* = 1312]
LDH (U/L)	221 (184–326) [*n* = 61]	210 (186–252) [*n* = 92]	199 (173–246) [*n* = 1159]	0.008 [*n* = 1312]
Myoglobin (µg/L)	—	—	90 (90–90) [*n* = 1]	Not applicable
PSA (µg/L)	0.94 (0.20–3.23) [*n* = 68]	1.10 (0.62–2.10) [*n* = 115]	1.40 (0.70–2.80) [*n* = 1338]	0.008 [*n* = 1521]
Uric acid (mmol/L)	0.38 (0.33–0.43) [*n* = 4]	0.42 (0.37–0.45) [*n* = 12]	0.34 (0.29–0.42) [*n* = 162]	0.11 [*n* = 178]

*Kruskall–Wallis test

**Fig. 1:**
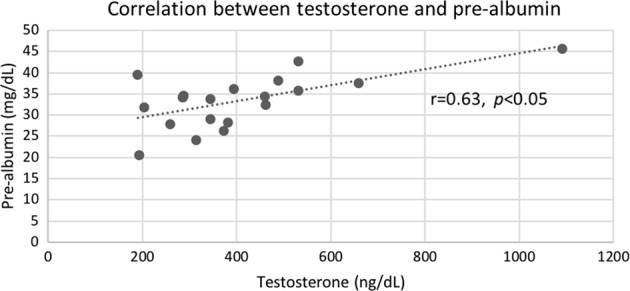
Correlation and trend line for testosterone versus pre-albumin levels (*n* = 19)

The results of the exploratory sub-study of pre-albumin are presented in Fig. 1. There was a moderate-positive association (*r* = 0.63) that was statistically significant (*p* < 0.05) between pre-albumin and testosterone levels.

## Discussion

Our results from the UPOD portion of this study indicate that lower levels of testosterone in adult men who are being assessed for possible hypogonadism are consistently associated with lower levels of estradiol, PSA, SHBG, and LH. In this study, associations between testosterone levels and LDH, BMI, BAP, and age appeared to be dependent on whether the men were older or younger than 56 years of age. The other non-hormonal biomarkers of muscle damage (CK), and metabolism (hemoglobin A1c and uric acid) showed either inconsistent or clinically insignificant associations with testosterone levels.

Notably, we observed a significant association between higher vitamin D levels and higher testosterone concentrations only in men older than 56 years of age. Previous studies have yielded conflicting results regarding associations between testosterone and vitamin D levels [15–17]. Our results therefore suggest the possibility that the age of subjects may play an important role in accounting for some of the variations in vitamin D levels that have been previously observed.

Creatinine levels demonstrated an interesting reverse association with testosterone levels between the younger and older men of our study. Among men younger than 56 years of age, normal testosterone was associated with higher levels of creatinine. Presumably this reflects the fact that creatinine is a surrogate marker of muscle mass [18], and testosterone stimulates muscle growth. In the older group of men (>56 years of age), the association seemed to be less pronounced and in the opposite direction (i.e., higher testosterone associated with lower creatinine). Since creatinine is also a marker of renal function, our results suggest the possibility that declining renal function could account for the higher levels of creatinine that were observed in the older men with lower testosterone levels. Thus, associations between creatinine levels and testosterone are likely to reflect a complex balance between muscle mass and renal function that varies with age.

The strong and consistent association between lower levels of LH in men with lower levels of testosterone in this study suggests that secondary hypogonadism was a more common etiology than primary hypogonadism in our study population. This finding is consistent with previous studies that have demonstrated that secondary hypogonadism accounts for ~80% of cases of adult men presenting for evaluation of late onset hypogonadism [19]. Hypogonadism is also frequently associated with obesity and other metabolic disturbances that can lower the level of SHBG [19, 20]. The latter association is consistent with the significantly lower levels of SHBG and higher BMI that we observed in this study among men with lower and lowest levels of testosterone. It should be noted, however, that we observed a significant association between BMI and testosterone only in the men who were older than 56 years of age, and even then, the magnitudes of the differences in median BMI were relatively modest.

The lower estradiol that we observed in men with low testosterone in our study is consistent with a previous report [21]. However, we extend this observation by also reporting a significant association between lower testosterone levels and higher BAP levels, primarily in men younger than 56 years of age. BAP is a marker of bone metabolism, and elevated levels in men have been associated with osteoporosis [22, 23]. Reduced estradiol concentrations have important clinical implications, given the critical role of estradiol on bone density, body composition, and sexual function in men [24–26]. In future studies, therefore, it will be important to correlate the results of bone densitometry to testosterone, BAP and estradiol levels. Although the available evidence is still inconclusive, we speculate that the concurrent presence of low testosterone, low estradiol, and high BAP could turn out to be a clue to the presence of osteoporosis in men younger than 56 years of age.

Median PSA values in this study were 40% higher for the low testosterone group compared with the lowest testosterone group (Table 1; 0.64 µg/L vs 0.90 µg/L). However, there was less than a 6% difference in PSA between the low testosterone (0.90 µg/L) and normal testosterone groups (0.95 µg/L), thereby suggesting a plateau that is consistent with a saturation effect [27]. These results and others demonstrate that PSA is exquisitely sensitive to androgen status, and low testosterone levels are associated with low PSA concentrations [28]. Maximal PSA concentration appears to be achieved at relatively low serum testosterone concentrations. Rastrelli et al. argued that a low PSA level in men older than 40 years suggested the presence of hypogonadism [28]. The presence of a low PSA level may thus provide additional biochemical evidence for systemic testosterone deficiency, which may be of clinical utility in making the diagnosis in borderline clinical cases.

The observation that LDH was elevated in older men with low testosterone was unexpected, and we are not aware of prior studies reporting this finding. LDH is present in all cells, and increased levels are a non-specific indicator of tissue damage, particularly muscle [29, 30]. Since low testosterone levels are associated with decreased muscle mass, we wonder whether this association reflects increased muscle breakdown in the low testosterone state. Our finding that LDH levels are higher in men with low testosterone therefore suggests that there may be value in measuring LDH in men suspected of having low testosterone.

The results from the exploratory, pre-albumin sub-study are intriguing because they provide preliminary evidence of a statistically significant (*p* < 0.05*)* trend towards a catabolic state in men with lower levels of testosterone. Because we included in this study only those men who were otherwise healthy and free of serious disease, we believe that this trend was not likely to be just an artifact due to other known causes of changes in transthyretin, such as severe inflammatory disease and malnutrition [31]. We acknowledge, however, that our sample size was relatively small. Our finding will therefore need to be replicated and extended in larger prospective studies. This is important because pre-albumin might have the potential to become an objective, biochemical marker for the catabolic state produced by low testosterone, thereby justifying consideration of therapy to reverse the catabolic state.

Our study has certain other limitations. Because contemporaneous measurements of testosterone and BMI were not available in some study subjects, and because we had only low numbers for some of the measured biomarkers, it was not statistically feasible to correct or adjust for BMI in the associations between the testosterone groups and the biomarkers. Despite this limitation, our study results confirmed and extended previously reported associations between elevated body mass index and low testosterone in men presenting to a tertiary academic medical center [32]

In the UPOD study, we did not control or adjust for the time of the blood draw for testosterone measurements or the general state of health of the study subjects. The timing of the blood draw is important in younger men, whose testosterone levels have diurnal variation, with levels peaking early in the morning [33], but younger men were not included in this study. In older men (>45 years of age) such as most of those in our study, however, the diurnal effect on testosterone appears to be much less significant [34–36]. It should also be noted that the men in the UPOD study were all outpatients who were being tested for hypogonadism. None were critically ill or hospitalized, and there is no a priori reason to suspect any bias in the timing of the blood draws or the general state of health between the groups of our study subjects. Hence, we believe that it is not likely that these factors could have accounted for the associations between biomarkers and testosterone that we observed in this study. Nevertheless, we acknowledge that there is still an open question of whether the associations that we observed have anything to do with low testosterone, or if the low testosterone just reflected general health and had no direct relationship to the markers of interest.

Our results, *in toto*, provide evidence supporting the concept that the effects of testosterone deficiency in men are far-ranging and involve numerous biological systems, as reflected in the broad, systemic set of biological changes that we observed in hormonal and non-hormonal biomarkers. We believe that alterations in these biomarkers may provide useful information that may enhance the accuracy and reliability of serum testosterone alone for the biochemical diagnosis of testosterone deficiency.

Whereas current clinical guidelines emphasize low values of total testosterone concentrations as a requirement for making the diagnosis of testosterone deficiency, it is acknowledged that there is no absolute testosterone concentration that accurately and reliably separates men who do and who do not have testosterone deficiency [37, 38] We believe the results of the present study support the possibility that a set of biomarkers can be used to assist with the identification of these men, including PSA, LDH, estradiol, creatinine, and possibly BAP. We also believe that this approach merits further exploration.

In conclusion, low testosterone levels are associated with significant changes in a variety of non-cardiovascular biomarkers. This finding may lead to improved laboratory diagnosis of men with low testosterone via objective evidence of physiologic changes produced by testosterone deficiency.
